# 9-DOF IMU-Based Attitude and Heading Estimation Using an Extended Kalman Filter with Bias Consideration

**DOI:** 10.3390/s22093416

**Published:** 2022-04-29

**Authors:** Sajjad Boorghan Farahan, José J. M. Machado, Fernando Gomes de Almeida, João Manuel R. S. Tavares

**Affiliations:** 1 Faculdade de Engenharia, Universidade do Porto, 4200-465 Porto, Portugal; sbfarahan@fe.up.pt; 2 Departamento de Engenharia Mecânica, Faculdade de Engenharia, Universidade do Porto, 4200-465 Porto, Portugal; jjmm@fe.up.pt (J.J.M.M.); fga@fe.up.pt (F.G.d.A.)

**Keywords:** gyroscope, accelerometer, magnetometer, sensor fusion, FFT diagrams, nonlinear, bias

## Abstract

The attitude and heading reference system (AHRS) is an important concept in the area of navigation, image stabilization, and object detection and tracking. Many studies and works have been conducted in this regard to estimate the accurate orientation of rigid bodies. In most research in this area, low-cost MEMS sensors are employed, but since the system’s response will diverge over time due to integration drift, it is necessary to apply proper estimation algorithms. A two-step extended Kalman Filter (EKF) algorithm is used in this study to estimate the orientation of an IMU. A 9-DOF device is used for this purpose, including a 6-DOF IMU with a three-axis gyroscope and a three-axis accelerometer, and a three-axis magnetometer. In addition, to have an accurate algorithm, both IMU and magnetometer biases and disturbances are modeled and considered in the real-time filter. After applying the algorithm to the sensor’s output, an accurate orientation as well as unbiased angular velocity, linear acceleration, and magnetic field were achieved. In order to demonstrate the reduction of noise power, fast Fourier transform (FFT) diagrams are used. The effect of the initial condition on the response of the system is also investigated.

## 1. Introduction

Attitude and heading estimation are two of the most important and interesting fields in the areas of navigation, autonomous vehicles, image stabilization, and object detection and tracking, just to cite a few. In this regard, the angular orientation of a rigid body in space is often referred to as attitude. An attitude and heading reference system (AHRS) is a combination of instruments that can estimate the rigid body’s attitude accurately.

Microelectromechanical system (MEMS) inertial sensors have become widely available in recent years, and this is because of their small size and low cost. In addition, because the inertial sensor measurements are obtained at high sampling rates, they have wide usage in the field of attitude and heading estimation. However, the estimations that are achieved by inertial sensors are accurate on a short time scale, but for longer time scales suffer from integration drift. To overcome this problem, inertial sensors are usually combined with additional sensors, such as magnetometers.

**Figure 1:**
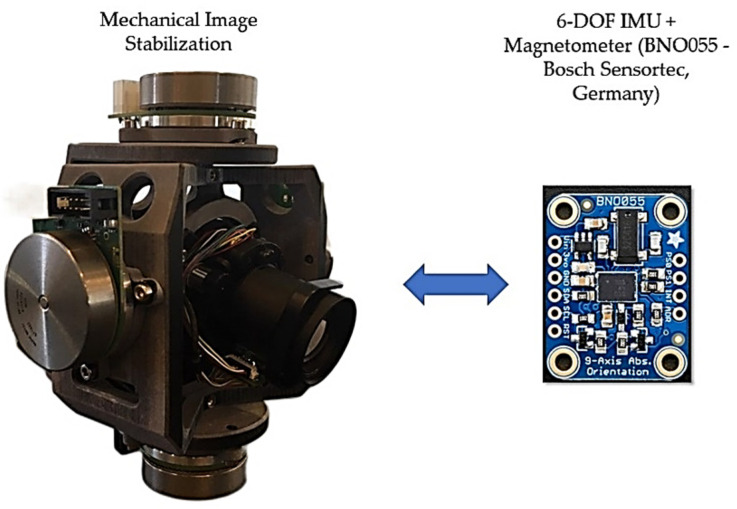
Example of an IMU with Magnetometer’s application (on the **left**) in Mechanical Image Stabilization; here, the sensor (on the **right**) is installed on the camera to detect its correct orientation.

Typically, the term inertial sensors are used to refer to the package which has a three-axis gyroscope and a three-axis accelerometer, and the package of these sensors is usually denoted as inertial measurement units (IMUs). Outstanding features of the IMUs are their low-cost, small size, and low power consumption. Therefore, IMUs can be widely used in any environment, such as indoor and outdoor applications. The sensor’s angular velocity, which denotes the rate of change of the sensor’s orientation, is measured with the gyroscope, and the external specific force acting on the sensor is also measured with the accelerometer. It should be noted that the accelerometer measurement contains the sensor’s acceleration and the earth’s gravity. It is clear that by integrating the gyroscope measurements one can achieve the orientation of the sensor, and by two times integration of the accelerometer measurements the pose of the sensor is achieved. The process of integrating the measurements from the gyroscope and accelerometer to obtain orientation and position information is often called dead-reckoning. However, in practice, many factors, such as sensor noise and bias, scale factor, sensor calibration, temperature, and alignment error, affect the accuracy and the performance of the IMUs. One of the IMU’s advantages consists of its application for image stabilization (Figure 1). Therefore, installing an IMU, equipped with a magnetometer on a camera, will allow the detection of its current orientation after exposure to external forces, removing the disturbances caused by them.

Common probabilistic methods for estimating an IMU’s orientation mostly rely on the ability to update the current orientation estimate using data provided by the IMU. For example, extended Kalman filtering (EKF) is one of the well-known algorithms widely applied in applications with the goal of state estimation. The EKF algorithm estimates unknown states using observations over time, which results in accurate orientation estimation. This filter can work in real-time and is designed for situations where the system’s dynamic is nonlinear.

Many works developed in the field of orientation estimation have used IMU measurements by considering sensor fusion algorithms, commonly based on Kalman filtering algorithms. Alandry et al. [1] used a five-axis IMU to reach proper orientation. Kulakova et al. [2] used an IMU, magnetometer, and the Global Navigation Satellite System (GNSS) to estimate orientation in addition to the position of an aerial vehicle. Other authors, such as Farhangian and Landry [3], used an IMU and magnetometer for their calibration technique for AHRS. Moreover, the authors applied an extended Kalman filter and PI controller. For the attitude estimation of a smartphone, Vertzberger and Klein [4] applied a two-stage adaptive complementary filter. Ludwig [5] aimed to determine accurate orientation estimation by using a genetic algorithm to tune the extended Kalman filter. Vitali and McGinnis [6] introduced a novel error-state Kalman filter to reach the proper orientation of an IMU. In another work, Bruschetta et al. [7] estimated the attitude of a motorcycle with a velocity-aided extended Kalman filter. Park et al. [8] estimated the orientation of a smartphone using a low-cost IMU by considering the magnetic disturbance effect. Jeon et al. [9] applied an IMU and a visually served paired system in a Kalman filtering framework to monitor high-speed structural movement. Jurman et al. [10] developed an algorithm for the calibration and alignment of an IMU with a magnetometer, which is employed for AHRS.

Auysakul et al. [11] used an IMU to estimate motion with the goal of video stabilization. Hence, the authors applied a Kalman filter to smooth the trajectory by eliminating the undesired motions. Munguía and Grau [12] developed an AHRS algorithm and used a direct configuration of EKF. That is, they used kinematic and error models to derive their extended Kalman filter algorithm. Setoodeh et al. [13] used three strapdown gyros to estimate attitude based on an EKF algorithm. In their work, the dynamic response of the inertial system is obtained without drift. For reaching the altitude, translational velocity, and orientation of a micro aerial vehicle, Zhong and Chirarattananon [14] used IMU in addition to monocular camera measurements. For the sensor fusion algorithm, they applied an iterated extended Kalman filter. Li and Wang [15] proposed an adaptive Kalman filter by utilizing linear models. They used a low-cost IMU equipped with a magnetometer to improve dynamic and computational efficiency. In another work, Deibe et al. [16] obviate the nonlinear behavior of attitude estimation by combining quaternions, state vector, and time-varying matrices. They also applied a classical Kalman filter approach in their algorithm.

One of the important issues in the orientation estimation is its parametrization, and in this article, unit quaternions were used for this goal. A unit quaternion uses a four-dimensional demonstration of the orientation and has an important advantage compared to the rotation matrix, where quaternions do not have any singularities. Young-Soo Suh [17] used a quaternion-based indirect Kalman filter for orientation estimation with an IMU, including a magnetometer for yaw angle estimation. For coping with uncertainty in attitude estimation in an IMU, Youn and Gadsden [18] presented a quaternion-based Kalman filter. Yong Ko et al. [19] used depth measurements and EKF for attitude estimation. So, they applied a quaternion-based representation instead of the usual Euler angle one. 

One of the key roles of using magnetometers in addition to IMUs is an accurate estimation of yaw angle. However, in most applications, the presence of ferromagnetic material in the vicinity of sensors made some disturbances in magnetometers. Roetenberg et al. [20] used an IMU with a magnetometer for orientation estimation of human body segments with a complementary Kalman filter. Again, Navidi and Landry [21] used a low-cost IMU with a magnetometer for orientation estimation by a complementary adaptive Kalman filter. In this work, the authors proposed an initialization method which, because of the nonlinear nature of the system, is one important step in attitude and heading determination. For improving the efficiency of orientation estimation, Fan et al. [22] considered the effect of magnetic disturbances in their work, which includes two parts, the first one is stationary state detection, and the second part is magnetic disturbance severity determination.

To have a good estimation regarding the position of rigid bodies, in addition to using IMUs, another sensor is usually necessary. Therefore, most researchers to have an accurate pose estimation use image cameras. Ligorio and Sabatini [23] used an IMU, a magnetometer, and camera system measurements. In principle, they used those sensors for fusion with two EKF for pose estimation of the moving camera. Alatise and Hancke [24] fused the measurements from a three-axis gyroscope, three-axis accelerometer, and a vision system to estimate the accurate position of a mobile robot. As in many works, an extended Kalman filter algorithm was used.

When IMUs are used in motion measurements, interesting advantages can be achieved, such as size reduction, low cost, and low power consumption. However, efficient and robust algorithms become necessary for acceptable performance. In this regard, for long-term pose and orientation estimation, the drift of IMUs results in significant accumulated errors. As a result, to improve the system performance, it is necessary to develop proper stochastic IMU error models and apply random noise minimization techniques. For instance, when an AHRS is used in applications with significant acceleration, attitude errors result, or the presence of ferromagnetic materials in the surrounding environment leads to errors in heading estimation.

The focus of this study was on the signal processing aspects of orientation estimation and on obtaining unbiased angular velocity, linear acceleration, and magnetic field using an inertial sensor and magnetometer. By now, many works have been developed related to orientation estimation with sensor fusion algorithms, which mostly use IMUs and Kalman filtering algorithms, but to the authors’ knowledge, the effect of gyroscope and accelerometer biases, as well as the effect of magnetometer bias in addition to ferromagnetic materials and magnetic disturbances at the same time, were not considered in released works. Therefore, to achieve optimized orientation and accurate unbiased angular velocity, linear acceleration, and magnetic field, an extended Kalman filtering (EKF) with 21 states was used. This includes three states for Euler angles, which demonstrate the orientation (pitch, roll, and yaw angles), nine states of unbiased angular velocity, linear acceleration, and unbiased and disturbance compensated magnetic field, and nine states of gyroscope, accelerometer, and magnetic field biases (each of them in *x*, *y*, and *z* directions). In addition, a two-step EKF algorithm was developed which uses the covariance inflation (CI) effect in the second step of the algorithm to optimize the results. Another important contribution of this article is the use of fast Fourier transform (FFT) diagrams to show the reduction of the power of noise. These kinds of diagrams are widely used for the investigation of the systems with nonlinear behaviors [25,26,27] and represent a novel way to show the noise reduction effect after applying the Kalman filtering algorithm in such systems. In addition, because the nonlinear systems are highly sensitive to correct initial conditions, at the end of the article, the effects of wrong initial conditions even in very small values are discussed.

This article consists of the following sections. Section 2 introduces quaternion-based EKF and magnetometers added to an IMU sensor. Section 3 describes the system configuration, and Section 4, presents the extended Kalman filtering (EKF) algorithm. In Section 5, the performance of the proposed method results is shown and discussed based on experimental tests. Finally, conclusions are given, and possible future research is suggested in Section 6.

## 2. Related Works

In order to achieve a reliable and accurate orientation estimation, it is common to use two or more different sensors. Therefore, using IMUs with magnetometers is usual, but they are characterized by uncertainties. Therefore, many studies have been developed to address these uncertainties and suggest robust sensor fusion algorithms. Thus, an efficient sensor fusion algorithm should include some features, e.g., offline calibration of IMU and magnetometer, online estimation of gyroscope, accelerometer, and magnetometer biases, adaptive strategies for surrounding ferromagnetic disturbances, and proper algorithm implementation for orientation estimation to reach accurate roll, pitch, and yaw angles.

In this study, the system input was obtained from a 6-DOF IMU plus Magnetometer device, which includes a three-axis gyroscope, a three-axis accelerometer, and a three-axis magnetometer. Moreover, for orientation, a quaternion-based extended Kalman filter (EKF) was developed.

Nazarahari and Rouhani [28] performed an experimental comparison among sensor fusion algorithms, such as various Kalman filters. These researchers published another article [29] which provides a survey of the state-of-the-art sensor fusion algorithms (SFAs) for orientation estimation. Additionally, Bancroft and Lachapelle [30] developed three SFA which mostly apply in pedestrian navigation. Feng et al. [31] combined IMU and Ultrawideband through an extended Kalman filter and unscented Kalman filter (UKF) for indoor positioning. Chang et al. [32] used the Global Positioning System (GPS) for position and IMU for velocity information. In fact, they fused that information with an indirect Kalman filter to reach accurate attitude estimation. Narasimhappa et al. [33] proposed a three-segment algorithm to evaluate the adaptive scale factor and applied their algorithm to control the drift error and random noise with a Sage-Husa adaptive Kalman filter. For attitude estimation, the algorithm developed by Yan et al. [34] detects external acceleration and then adjusts the noise variance of EKF to compensate for external acceleration. Benzerrouk and Nebylov [35] used IMU and ultrawideband in a direct Kalman filtering approach to pedestrian navigation. Liu et al. [36] proposed an extended Kalman filtering framework for displacement and its uncertainties estimation with an IMU. Poulose et al. [37] used smartphone sensors for investigating five SFAs in terms of root mean square error and cumulative distribution functions, where most of the used algorithms are based on Kalman filtering. Wang et al. [38] developed a two-stage Kalman filter for object tracking, which uses a learning-based adaptive unscented Kalman filter for reducing the position estimation error. Again, in the area of applying learning methods, Kuzdeuov et al. [39] used a deep neural network in combination with an EKF for pose estimation. Potokar et al. [40] used doppler velocity log and IMU sensors in their developed algorithm and named it as: Invariant Extended Kalman filter.

Numerous works considered the magnetometers for reaching accurate orientation estimation. However, in many of them, the lack of considering the effect of ferromagnetic materials in the vicinity of a magnetometer is obvious. Actually, ferromagnetic materials or other magnetic fields near the sensors disturb the local earth magnetic field which can be measured with magnetometers.

Sabet et al. [41] considered the gyroscope bias and magnetic disturbances effect by using a low-cost IMU and EKF for orientation estimation. Evren et al. [42] introduced a master–slave Kalman filter, which includes an EKF and an Φ-algorithm with an IMU and a magnetometer to reach Euler angles, velocity, and acceleration. Zhao and Wang [43] used ultrasonic, IMU, and magnetometer sensors for orientation and displacement estimation. In their work, an EKF uses ultrasonic sensor data for position estimation and uses magnetometer and IMU data for orientation estimation. Tong et al. [44] developed an algorithm that uses a multiplicative extended Kalman filter and a Markov model for attitude estimation. So, the authors used an IMU with a magnetometer for this aim. Feng et al. [45] developed a linear Kalman filter by consideration of magnetic distortion effects for orientation estimation. Liu et al. [46] also used a 9-DOF device, including an IMU and a magnetometer, to estimate the orientation and applied an adaptive complementary filter for orientation estimation, which used an EKF for fusing prior information. Fan et al. [47] investigated the effects of magnetic disturbances on orientation estimation. Moreover, they developed four SFAs that contain gradient descent, explicit complementary, dual-linear Kalman, and EKF.

## 3. System Configuration

The Kalman filter is an estimator that estimates the state of a dynamic linear or nonlinear system affected by noise. So, it uses measurements that are linear or nonlinear functions of the system state where it is affected by additive noise. Hence, two critical variables in Kalman filtering are the mean and the covariance of the distribution. This is commonly called a filter because, basically, it separates the “signals” from “noise” and uses observations up to and including the time that the state of the dynamic system is to be estimated.

### 3.1. Inertial Sensors

In this study, a 9-DOF device, which contains a 3-axis gyroscope, a 3-axis accelerometer, and a 3-axis magnetometer, was used. MEMS gyroscopes are designed for angular velocity measurements. However, they are reliable just for short-time calculations. These sensors suffer from a small offset due to temperature effects, which introduce large integration errors in long-time usage. In this regard, inclination information from the accelerometer can be used to correct the gyroscope’s drift. Since accelerometers cannot detect rotations about the vertical axis, magnetic sensing can be added. The magnetometer is sensitive to the earth’s magnetic field and can thus be used to correct the drift of the gyroscope about the vertical axis.

### 3.2. Coordinate Frames

For better understanding, here, the used coordinate frames are introduced.

The Body Frame, b, is used for moving IMU whose origin is in the center of the accelerometer;

The Navigation Frame, n, is used to specify the b-frame relative to it;

The Inertial Frame, i, where the gyroscope and accelerometer measurements are obtained, and its origin is located at the center of the earth;

The Earth Frame, e, coincides with the inertial frame but rotates with the earth.

It should be noted that superscripts are used here to indicate in which coordinate frame a vector is expressed, and double superscripts are used to indicate from which coordinate to which coordinate frame the rotation is defined.

### 3.3. Probabilistic Models

The nonlinear nature of the orientation makes its estimation complex. It is known that when the distribution is Gaussian, the conditional probability distribution can be defined by its mean and covariance.

An assumption in the developed algorithm is that it follows the Markov property, which implies that all information up to the current time is contained in the current state $x_{t}$.

The nonlinear model of the state and measurements can be modeled by having the process noise $\left(w_{t}\right)$, which is zero-mean Gaussian with covariance Q, and $w_{t}\;∼\;N(0\;,\;Q)$, and the measurement noise $\left(e_{t}\right)$, where $e_{t}\;∼\;N(0\;,\;R)$ and R is its covariance.

### 3.4. Orientation Parametrizing

For orientation parametrizing, four methods were used in the developed algorithm. It should be noted that detailed equations are given and explained in Appendix A.

#### 3.4.1. Rotation Matrices

The rotation matrix is an orthogonal matrix, and if two coordinate frames u and v are considered, then the rotation matrix will rotate a vector x from v-frame to u-frame as:

(1) $$x^{u}=R^{u\;v}x^{v}\;\;x^{v}={\left(R^{u\;v}\right)}^{T}x^{u}=R^{v\;u}x^{u}$$

#### 3.4.2. Rotation Vector

The rotation between two coordinates can be expressed by combining an angle α and a unit vector n, which leads to a rotation vector η=n α.

#### 3.4.3. Euler Angles

**Figure 2:**
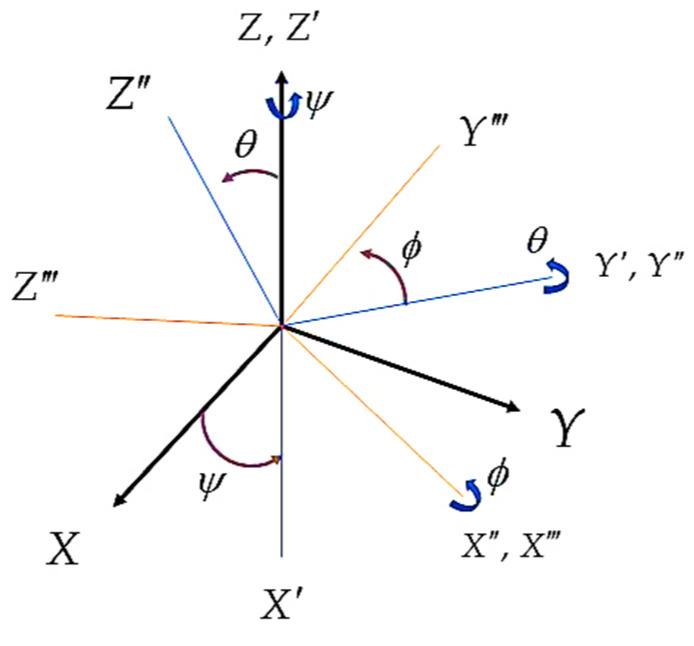
Rotation between frames using Euler Angles.

Rotation can also be defined with Euler angles. So, first the yaw angle (ψ) rotation around the z-axis, then the pitch angle (θ) around the y-axis, and finally the roll angle (ϕ) around the x-axis are used, leading to the rotation matrix (Figure 2). Often, yaw angle is referred to as heading, while roll and pitch angles together are referred to as inclination.

#### 3.4.4. Unit Quaternions

The last, but not the least rotation format of orientation parametrization is using unit quaternion, which is a 4-D expression of orientation, and a rotation can be defined using unit quaternions as:

(2) $${\bar{x}}^{u}=q^{u\;v}⊙{\bar{x}}^{v}⊙{\left(q^{u\;v}\right)}^{c}$$

In order to see complementary relations, please refer to Appendix A. 

### 3.5. Linearization

Special orthogonal group SO(3) is the group of rotations in 3-D. Hence, one can exchange the orientation $q_{t}^{nb}$ in terms of linearization point as a unit quaternion ${\tilde{q}}_{t}^{nb}$ or as a rotation matrix ${\tilde{R}}_{t}^{nb}$, and a rotation vector $\eta_{t}$ that has a small value:

(3) $$q_{t}^{nb}=\exp_{q}\left(\frac{\eta_{t}^{n}}{2}\right)⊙{\tilde{q}}_{t}^{nb},\;R_{t}^{nb}=\exp_{R}\left(\eta_{t}^{n}\right){\tilde{R}}_{t}^{nb}$$

### 3.6. Measurement Models

As aforementioned, the IMU’s measurements are corrupted by noise, which is quite Gaussian. In addition, the measurements are biased.

#### 3.6.1. Gyroscope Measurement Model

The gyroscopes measurement $y_{g\;}$ is the sum of the angular velocity ω, the slow varying bias term $x_{g}$, and a white noise term $v_{g}$:

(4) $$\begin{array}{l} y_{g,t}=\omega_{t}^{b}+\;x_{g,t}^{b}+v_{g,t}^{b}\\ x_{g,t+1}=(1-\lambda_{x_{g}}T)\;x_{g,t}+v_{x_{g},\;t} \end{array}$$

where $\lambda_{x_{g}}$ is a correlation time factor and $v_{x_{g},\;t}$ is white Gaussian noise.

#### 3.6.2. Accelerometer Measurement Model

The accelerometer measurement $y_{a}$ is the sum of the linear acceleration a, the earth’s gravity g, the bias $x_{a}$, which is modeled as a first-order low-pass filter, and a white noise term $v_{a}$:

(5) $$\begin{array}{l} y_{a,t}=R_{t}^{bn}\left(a_{t}^{n}-g^{n}+x_{a\;,\;t}\right)+v_{a,t}\\ x_{a,t+1}=(1-\lambda_{x_{a}}T)\;x_{a,t}+v_{x_{a},\;t} \end{array}$$

Again, $\lambda_{x_{a}}$ is a correlation time factor and $v_{x_{a},t}∼N\left(0,\;\sum_{x_{a}}\right)$.

#### 3.6.3. Magnetometer Measurements Model

The magnetometer signals are the sum of the earth’s magnetic field vector m, a disturbance vector $x_{m}$, and a white noise term $v_{m}$:

(6) $$\begin{array}{l} y_{m,\;t}=R_{t}^{bn}\left(\left(1-\Gamma^{2}\right)m_{t}^{n}+x_{m\;,\;t}\right)+v_{m,t}\\ x_{m,t+1}=(1-\lambda_{x_{m}}T)\;x_{m,t}+v_{x_{m},\;t} \end{array}$$

As explained previously, external magnetic fields and ferromagnetic materials near the used sensor change the real magnetic flux. Although the soft-iron effects are compensated with a calibrated magnetometer, the effect of ferromagnetic material and weakening effects (Γ) should be considered in the output measurements of the magnetometer. It should be noted that the weakening effect is due to the surrounding ferromagnetic infrastructure.

**Figure 3:**
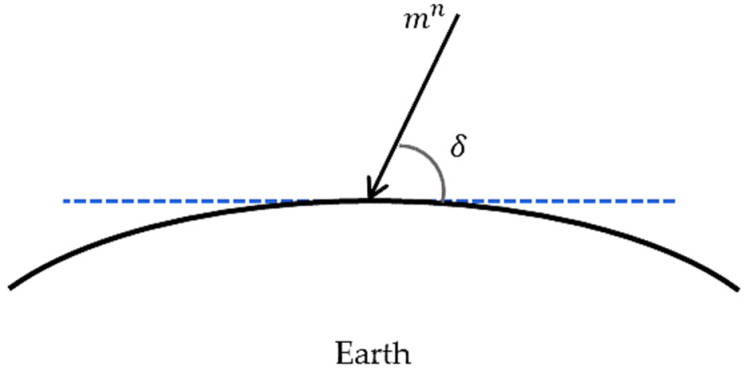
Schematic representation of the Dip angle (the blue dashed line refers to the horizontal component of the magnetometer location).

In order to calculate the earth’s magnetic field $m^{n}$, one should know the dip angle δ which demonstrates the ratio between the horizontal and vertical components of the magnetometer’s location (Figure 3). This angle in the experiment location (Porto) is 53° and:

(7) $$m^{n}={\left(0\cos\;\delta \;-\;\sin\;\delta \right)}^{T}$$

### 3.7. State Selection and Its Dynamic

The relationship between the orientation and angular velocity is described as:

(8) $$\frac{dq^{nb}}{dt}=q^{nb}⊙\frac{1}{2}{\bar{\omega }}_{nb}^{b}$$

Now, using unit quaternion, one can obtain:

(9) $$q_{t+1}^{nb}=q_{t}^{nb}⊙\exp_{q}\left(\frac{T}{2}\omega_{nb,t}^{b}\right)$$

To achieve the goal of this study, the following 21 × 1 state system $\hat{x}$ was considered:

(10) $${\hat{x}}_{t}={\left[\begin{array}{ccccccc} {\left(\eta_{t}^{n}\right)}^{T} & {\left(\omega_{t}^{b}\right)}^{T} & {\left(x_{g,t}^{b}\right)}^{T} & {\left(a_{t}^{b}\right)}^{T} & {\left(x_{a,t}^{b}\right)}^{T} & {\left(m_{t}^{b}\right)}^{T} & {\left(x_{m,t}^{b}\right)}^{T} \end{array}\right]}^{T}$$

where $\eta^{n}$ is the rotation vector that is used for calculating the orientation, i.e., the roll, pitch, and yaw angles, $\omega^{b}$ is the bias-compensated (unbiased) rotation velocity of the body, $x_{g}$ is the bias of gyroscope, $a^{b}$ is the unbiased linear acceleration of the body, $x_{a}$ is the bias of accelerometer, $m^{b}$ is the unbiased magnetic field, and $x_{m}$ is the bias of magnetometer.

### 3.8. Initial Conditions

A common approach adopted to determine the initial orientation is to use the first accelerometer and magnetometer samples [48].

#### State Initialization

During the initial period of simulation, where $\left\|y_{a}\right\|\approx g$, one can calculate $\bar{g}=\frac{1}{T}\int_{0}^{T}-y_{a}\left(t\right)\;dt$, and by denoting $\bar{g}={\left[{\bar{g}}_{1},\;{\bar{g}}_{2},\;{\bar{g}}_{3}\right]}^{T}$, and initial roll and pitch values can be computed as:

(11) $$\begin{array}{l} \hat{\phi }\left(T\right)=a\tan2\left({\bar{g}}_{2},\;{\bar{g}}_{3}\right)\\ \hat{\theta }\left(T\right)=a\tan2\left(-{\bar{g}}_{1},\;\sqrt{{\bar{g}}_{2}^{2}+{\bar{g}}_{3}^{2}}\right) \end{array}$$

From the magnetometer’s measurement in combination with the above initial roll and pitch angles, one can obtain the initial yaw angle:

(12) $$\begin{array}{c} {\bar{m}}^{w}=\;\left[\begin{array}{ccc} \cos\left(\theta \right) & \sin\left(\theta \right)\;\sin\left(\phi \right) & \sin\left(\theta \right)\;\cos\left(\phi \right)\\ 0 & \cos\left(\phi \right) & -\sin\left(\phi \right)\\ -\sin\left(\theta \right) & \cos\left(\theta \right)\;\sin\left(\phi \right) & \cos\left(\theta \right)\;\cos\left(\phi \right) \end{array}\right]\;{\bar{m}}^{b}\\ {\bar{m}}^{b}=\frac{1}{T}\int_{0}^{T}y_{m}\left(t\right)\;dt \end{array}$$

(13) $$\hat{\psi }\left(T\right)=a\tan2\left(-{\bar{m}}_{2}^{w},\;{\bar{m}}_{1}^{w}\right)$$

Now, one can estimate the initial rotation matrix $R^{bn}$ (see Appendix A).

During the same initialization period, while the IMU is assumed to be stationary, the first measurements of the gyroscope and accelerometer can be averaged to provide initial estimates of their biases.

## 4. Extended Kalman Filtering Algorithm

**Figure 4:**
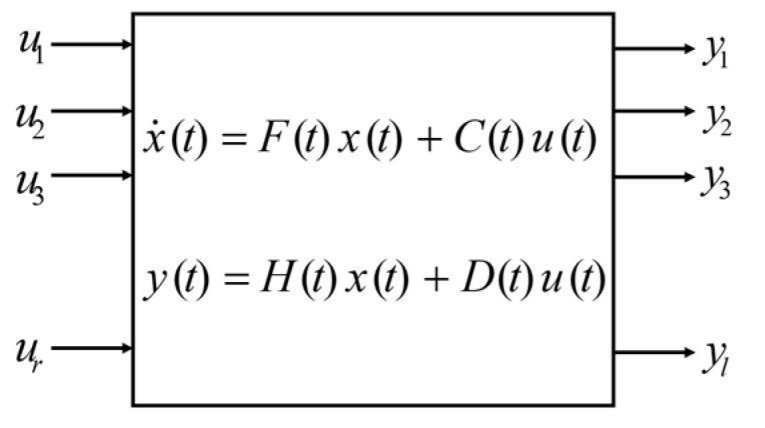
Block diagram of a dynamic system: $u_{i}$ is the control inputs, $x_{i}$ the internal state variable, and $y_{i}$ is the output, which is called observations or measurements.

The extended Kalman filter (EKF) is the extended version of Kalman filtering which makes use of a nonlinear state-space model (Figure 4).

The algorithm performs a time and a measurement update, and for time update one has:

(14) $$\begin{array}{l} {\hat{x}}_{t+1\;|\;t\;}=\;f_{t}\;\left({\hat{x}}_{t\;|\;t},\;u_{t}\right)\\ P_{t+1\;|\;t}=F_{t}\;P_{t\;|\;t}\;F_{t}^{T}\;+\;G_{t}\;Q\;G_{t}^{T} \end{array}$$

(15) $$F_{t}\;=\;\frac{\partial \;f_{t}\;\left(x_{t},\;u_{t},\;w_{t}\right)}{\partial \;x_{t}},\;G_{t}\;=\;\frac{\partial \;f_{t}\;\left(x_{t},\;u_{t},\;w_{t}\right)}{\partial \;v_{t}},\;(Cond.\;w_{t}\;=\;0,\;x_{t}={\hat{x}}_{t\;|\;t})$$

where P is the state covariance, and ${\hat{x}}_{t+1\;|\;t}$ means that the state estimate at time t+1 given measurement is up to time t, and Q is the measurement noise of the IMU, which can be derived using the process noise covariance matrix.

In the measurement update, one has:

(16) $$\begin{array}{l} {\hat{x}}_{t\;|\;t}\;=\;{\hat{x}}_{t\;|\;t-1}\;+\;K_{t}\;\varepsilon_{t}\\ P_{t\;|\;t}\;=\;P_{t\;|\;t-1}\;-\;K_{t}\;S_{t}\;K_{t}^{T} \end{array}$$

with:

(17) $$\varepsilon_{t}=\;y_{t}\;-\;{\hat{y}}_{t\;|\;t-1},\;S_{t}=H_{t}\;P_{t\;|\;t-1}\;H_{t}^{T}+R,\;K_{t}=P_{t\;|\;t-1}\;H_{t}^{T}\;S_{t}^{-1}$$

(18) $${\hat{y}}_{t\;|\;t-1}=\;h\;\left({\hat{x}}_{t\;|\;t-1}\right),\;H_{t}=\;\frac{\partial \;h_{t}\;\left(x_{t}\right)}{\partial \;x_{t}}\;(Cond.\;x_{t}={\hat{x}}_{t\;|\;t-1})$$

### Orientation Estimation

An EKF is used to parametrize orientation around a linearization point in terms of quaternions, and in this algorithm, $\eta_{t}^{n}$ is the state vector [49]:

(19) $$\begin{array}{ll} \eta_{t+1}^{n} & =f_{t}\left(\eta_{t}^{n},y_{g,t},\;x_{g,t},\;v_{g,t}\right)\\ & =2\log\left(\exp_{q}\left(\frac{\eta_{t}^{n}}{2}\right)⊙{\tilde{q}}_{t}^{nb}⊙\exp_{q}\left(\frac{T}{2}\left(y_{g,t}-x_{g,t}-v_{g,t}\right)\right)⊙{\tilde{q}}_{t+1}^{bn}\right) \end{array}$$

(20) $${\tilde{q}}_{t\;|\;t}^{nb}=\exp_{q}\left(\frac{{\hat{\eta }}_{t}^{n}}{2}\right)⊙{\tilde{q}}_{t\;|\;t-1}^{nb}$$

(21) $$\omega_{t+1}^{b}=y_{g,t}-x_{g,t}-v_{g}$$

(22) $$x_{g,t+1}=\left(1-\lambda_{x_{g}}T\right)x_{g,t}+v_{x_{g}}$$

(23) $$a_{t+1}^{b}=y_{a,t}+R_{t}^{bn}\left(g^{n}-x_{a,t}\right)-v_{a}$$

(24) $$x_{a,t+1}=\left(1-\lambda_{x_{a}}T\right)x_{a,t}+v_{x_{a}}$$

(25) $$m_{t+1}^{b}=\left(\frac{1}{1-\Gamma^{2}}\right)\left(y_{m,t}-R_{t}^{bn}\left(x_{m,t}\right)-v_{m}\right)$$

(26) $$x_{m,t+1}=\left(1-\lambda_{x_{m}}T\right)x_{m,t}+v_{x_{m}}$$

At every step k, the system state $\hat{x}$ is updated.

Jacobian F is a dynamic coefficient matrix formed by the partial derivatives of the nonlinear prediction model Equations (19)–(26) with respect to the system state $\hat{x}$. Similarly, Jacobian G is formed by the partial derivatives of the prediction model with respect to the system inputs. Moreover, Jacobian $H_{t}$ is the measurement sensitivity matrix and is defined by the partial derivatives of the measurement prediction model $h\;\left(\hat{x}\right)$ with respect to the system state $\hat{x}$.

Using Equation (16), the innovation matrix S is calculated in the first step of the algorithm, and in the second step of the algorithm, the covariance inflation (CI) effect is considered on it to reach optimized results [50]. Moreover, in Equation (16), K, which is the Kalman gain, should be updated.

Now, the state-space vector is updated, and as the final step, the linearization point is updated as re-linearization, which updates ${\tilde{q}}_{t\;|\;t-1}^{nb}$ to ${\tilde{q}}_{t\;|\;t}^{nb}$ (Equation (20)).

**Figure 5:**
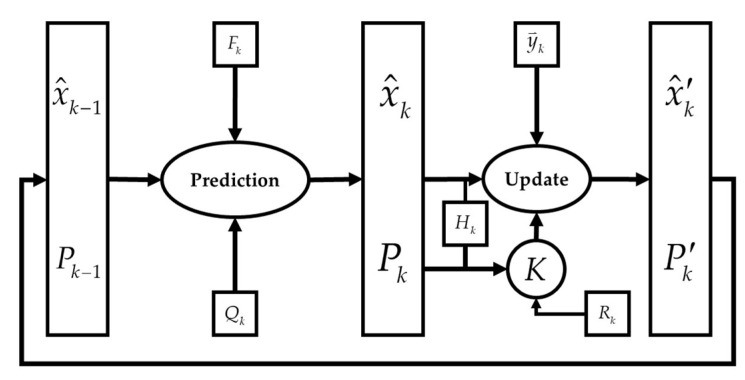
Flowchart of the Extended Kalman Filter.

The general flowchart of the extended Kalman filter (EKF) is depicted in Figure 5.

## 5. Results and Discussion

In this section, the results of extended Kalman filtering after applying its algorithm to the IMU’s outputs are presented and discussed. It should be noted that the desired algorithm was implemented using the MATLAB platform.

**Figure 6:**
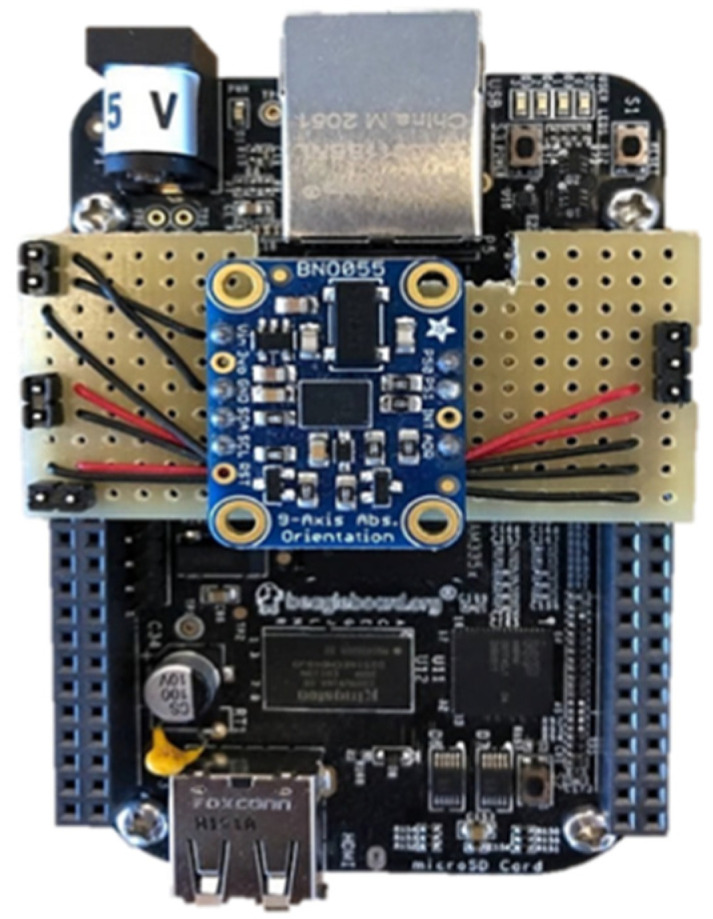
Used 6-DOF IMU with Magnetometer: model BNO055 from Bosch Sensortec (Germany).

As aforementioned, all the experiments were conducted with a 9-DOF IMU, which includes a three-axis gyroscope, a three-axis accelerometer, and a three-axis magnetometer. The used IMU model was the BNO055 unit (Bosch Sensortec, Reutlingen, Germany) (Figure 6).

In order to discuss the results of using extended Kalman filtering in IMU, two series of IMU outputs were investigated. One bunch is for the stationary IMU, which means the IMU is located on a table without any movement, and the second bunch is for the moving IMU where it is moved in random directions, and the data are collected as input for the developed EKF algorithm.

The frequency of the used IMU is 100 Hz. Then, the time interval between the samples was 0.01 s. First, the results for the stationary IMU are presented.

**Figure 7:**
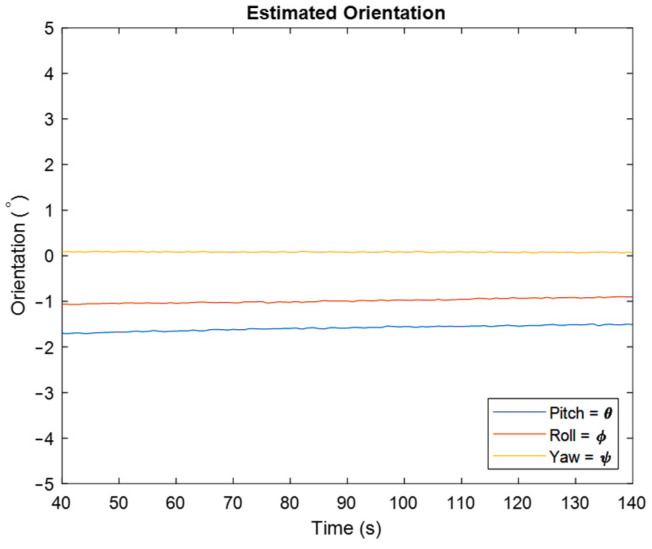
Estimated orientation for the stationary IMU by representing roll, pitch, and yaw angles.

In Figure 7, one can see the estimated orientation in degree for pitch, roll, and yaw angles. As is clear, the estimated orientation shows that the IMU is in a stationary situation, which confirms that the developed algorithm works properly. As aforementioned, one of the important issues in orientation estimation is estimating yaw angle, because accelerometers cannot detect rotations about the vertical axis. Therefore, magnetometers that are sensitive to the earth’s magnetic field are added to IMUs to correct the gyroscope’s drift in this direction. The result of using the magnetometer is clear from the shown estimated yaw angle.

**Figure 8:**
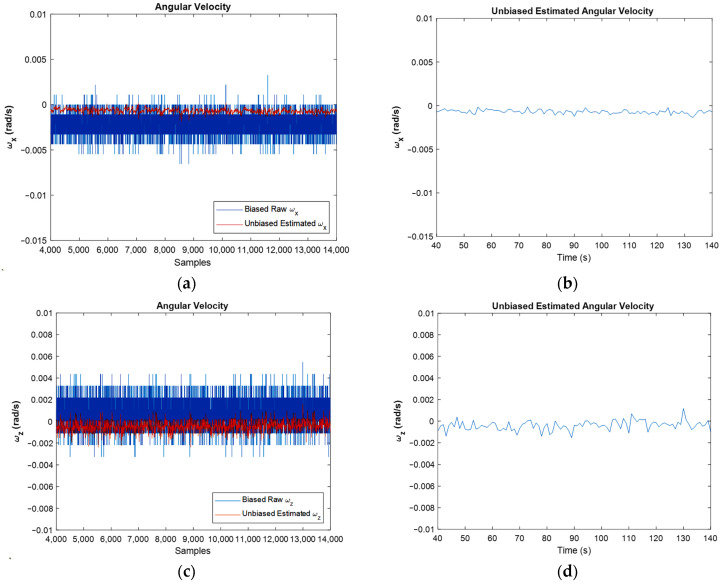
Biased raw angular velocity against estimated angular velocity for the whole samples (**a**,**c**), and for last amounts of estimation (**b**,**d**), in x and z directions, respectively.

In Figure 8, the width of the unbiased graphs is less than the width of the raw graphs. Moreover, the bias compensation is well visible. In this figure, the biased raw angular velocities, against unbiased ones for all samples and for the simulation period, are depicted. One of the main goals of this article is to eliminate the bias of sensors. Therefore, these graphs show that the bias of the gyroscope is compensated properly.

From Figure 8, which depicts the angular velocities in *x* and *z* directions, it is clear that the width of the graph in the unbiased figures is less than the biased one which shows the reduction in noise’s power, and this is demonstrated in the FFT diagrams clearly. Figure 8b,d depict the unbiased angular velocities in the time domain. It should be noted that in each second, 100 samples were read from IMU, and all of them were used in the EKF algorithm. However, in these two figures, aiming at simplicity, just the last results in each second are represented.

**Figure 9:**
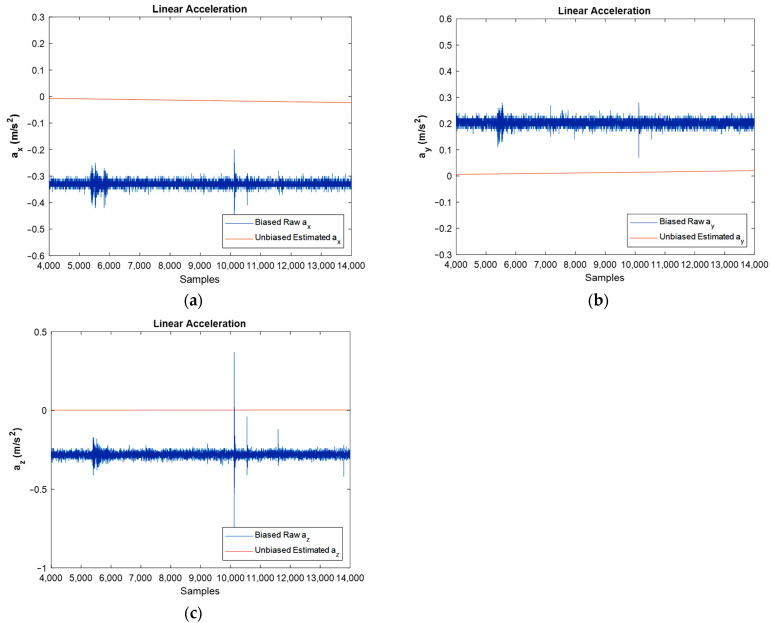
Estimated unbiased linear acceleration for stationary IMU in x, y and z directions against biased raw one: (**a**–**c**), respectively.

The same comparison was made regarding the linear acceleration of IMU. Therefore, the output of the accelerometer, which is the sum of the linear acceleration, earth’s gravity, and bias, were passed through the extended Kalman filter’s algorithm, and after reducing the earth’s gravity constant and eliminating the bias magnitude, linear acceleration was obtained as shown in Figure 9.

In Figure 9, the biased and filtered unbiased linear acceleration of stationary IMU can be observed. Because the IMU is fixed and does not have any movement, the IMU should show approximately zero acceleration in the three axes. However, there is some difference between raw data and filtered data and the main reason for this is the accelerometer’s bias or offset. However, after sensor fusion in the EKF algorithm, the bias magnitude is considerably eliminated.

Considering both the accelerometer bias and the compensation of its effect by a proper algorithm has a significant effect on both the orientation and pose estimation. In addition to correcting the gyroscope’s drift for orientation estimation, the accelerometer is used for position estimation by two-time integration. The main objective of this study was orientation estimation, but in future studies, pose estimation will be considered and accelerometer bias compensation will be effective.

Now is the time to discuss the earth’s magnetic field which is measured with the magnetometer, and the goal was to investigate the effect of EKF’s algorithm on it in order to remove the caused magnetic disturbances.

**Figure 10:**
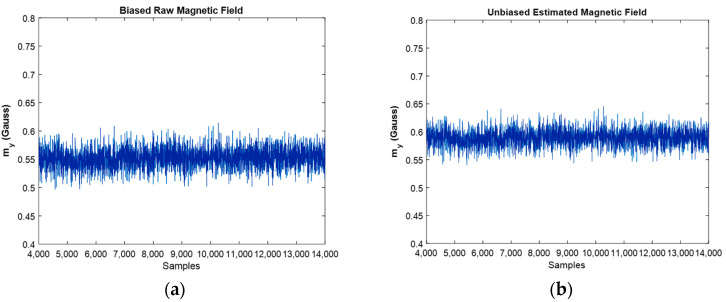
Biased raw magnetic field (**a**) against the estimated magnetic field in y direction (**b**).

Figure 10 depicts the comparison between biased, and unbiased magnetic fields in y direction. As it is clear from this figure, the value of magnetic disturbances was eliminated, and the designed algorithm successfully eliminated the effect of ferromagnetic materials in the vicinity of the magnetometer. In the experimental environment, due to the presence of ferromagnetic materials such as iron and the presence of electrical wires, magnetic disturbance occurs and affects the results. The unbiased magnetic field compensated the sum of the mentioned disturbances as well as the magnetometer’s bias effects, and this is the cause for the difference between the two graphs. In addition, regarding the location of the experiment (Porto city, Portugal) where the earth’s magnetic field is almost 53°, the unbiased magnetic field that compensates for the effects of the magnetic disturbance can be recognizable from the graph.

**Figure 11:**
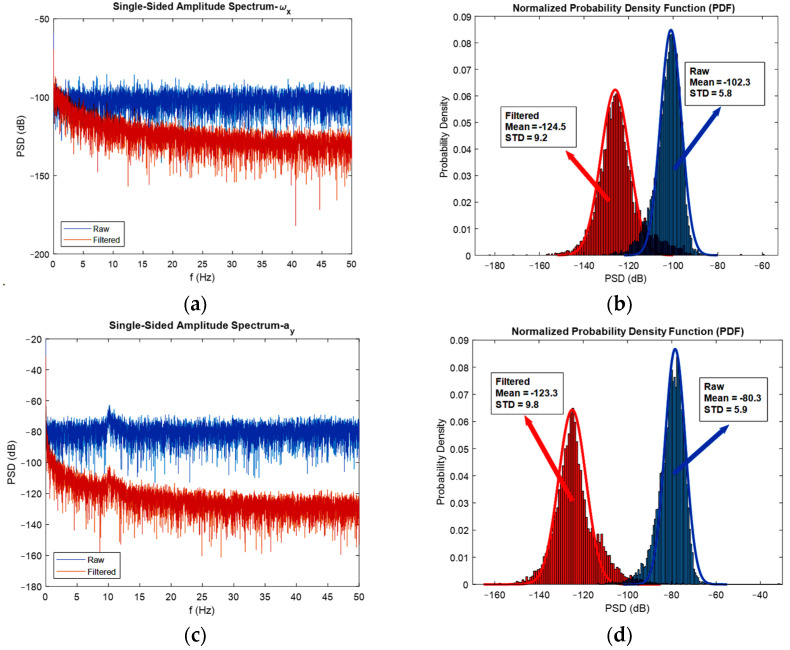

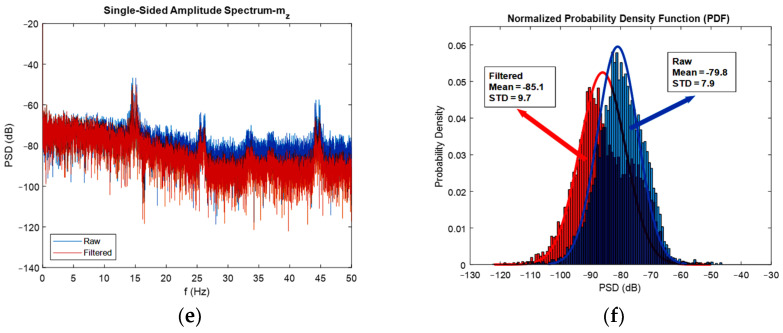
FFT diagrams (**a**,**c**,**e**), and histograms for the stationary gyroscope, accelerometer, and magnetometer (**b**,**d**,**f**), respectively.

As already mentioned, to realize the effect of extended Kalman filters on the power of noise, it is convenient to use FFT diagrams. In Figure 11, the FFT diagrams are shown for one direction of each angular velocity, linear acceleration, and magnetic field, respectively, to show how much of the noise’s power is reduced after the adopted sensor fusion strategy. In addition to the FFT diagrams, histograms are used to better clarify the issue of noise reduction.

As depicted in the FFT diagrams and histograms of Figure 11, the filter reduced the noise flow, especially in the high frequencies, i.e., by increasing the frequency, noise-cancelling phenomena increased. For instance, the mean of the noise power in the gyroscope was reduced by 25 dB, in the accelerometer reduced by 45 dB, and in the magnetometer, the noise flow decreased by 5 dB. Therefore, one can confirm the noise cancelation phenomenon successfully achieved by the developed extended Kalman filter. Another interesting thing that can be realized from the FFT diagrams and histograms is that the mean magnitude of the noise’s power is constant, and this exactly satisfies the assumption made regarding noise, which was considered white noise. Moreover, it can be seen from the histograms that the measurement noise resembles a Gaussian curve. One contribution of this study was the application of FFT diagrams and histograms to illustrate the reduction in noise power. In fact, plotting these kinds of graphs, especially frequency domain diagrams, represents a good method for investigating the effect of the developed filter on noise reduction.

The following results are for moving IMU, and first, the orientation estimation is discussed. 

**Figure 12:**
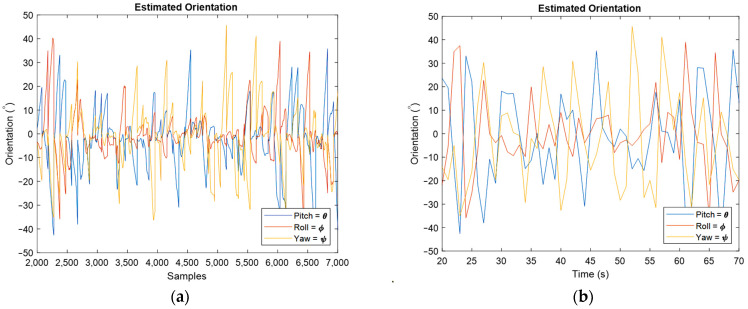
Estimated orientation for moving IMU by representing roll, pitch, and yaw angles for all samples (**a**), and for the last estimated value in each sampling rate (**b**).

**Figure 13:**
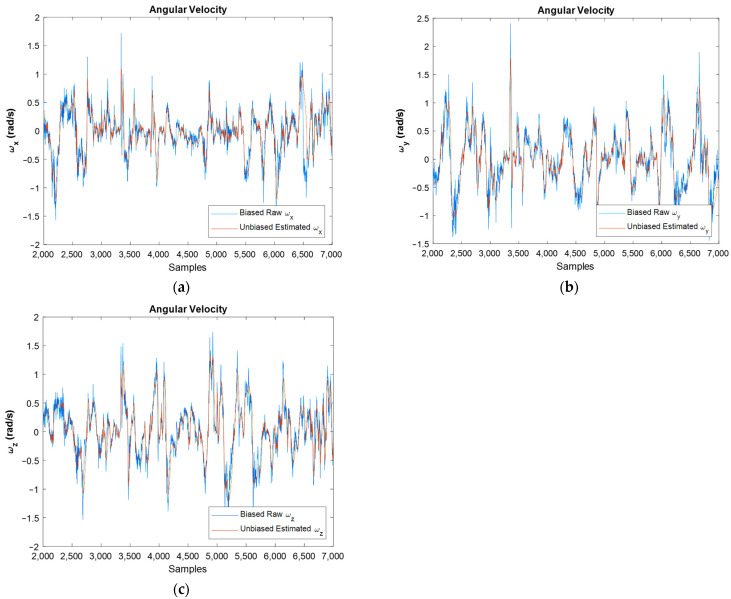
Estimated unbiased angular velocity for x, y, and z direction against raw ones: (**a**–**c**), respectively.

The obtained orientation estimation for moving IMU can be seen in Figure 12, where Figure 12a is for whole samples and Figure 12b concerns the estimated orientation from the final point of each sampling rate. The obtained angular velocity estimation is shown in Figure 13.

**Figure 14:**
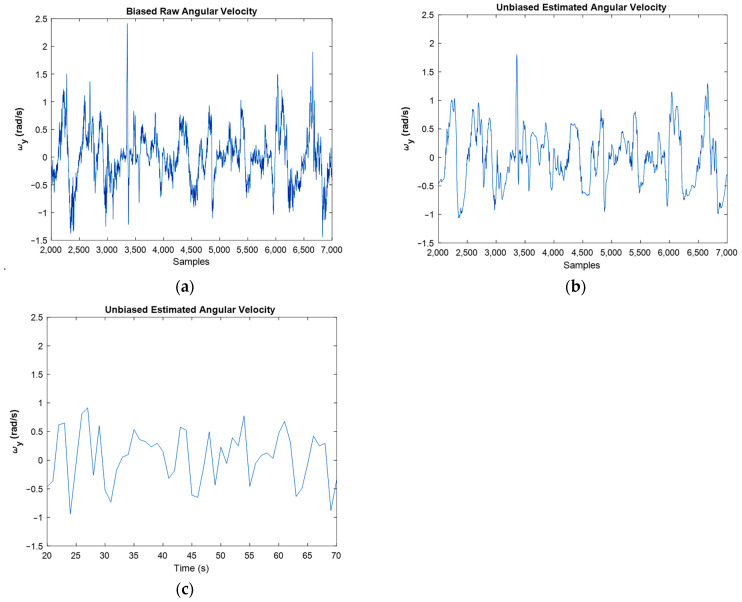
Raw angular velocities (**a**), against estimated angular velocities for samples (**b**), and for last amounts of estimation (**c**).

Figure 13 shows the biased raw angular velocities against unbiased filtered ones for moving IMU. One can see in this figure that the filter worked properly because filtered graphs follow the raw graphs, but the bias and noise were successfully eliminated. For an easier interpretation, Figure 14 presents the angular velocities separately. 

In Figure 14, the filtered plot followed the raw plot properly. However, the noise was reduced dramatically, and the bias was eliminated. One point in Kalman filtering is that this filter cannot follow the abrupt and very fast variations, so its estimations of these situations are very sensibly. However, for example, in Figure 14a, an abrupt change in the angular velocity happened around the 34th second (3400th sample). Yet, the implemented filter was able to estimate it properly (Figure 14b). 

**Figure 15:**
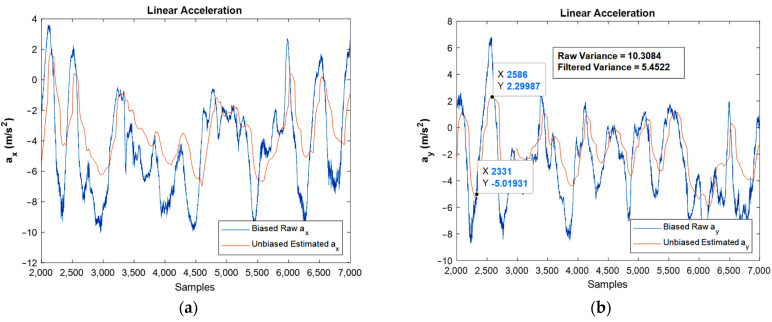
Estimated unbiased linear acceleration of moving IMU in x, and y directions against biased raw ones: (**a**,**b**), respectively.

Figure 15 shows the results regarding linear acceleration, which confirms the good performance achieved by the developed filter. 

Again, the bias compensated graphs in Figure 15 followed the path of linear acceleration properly and eliminated the bias effect. In addition, the noise was reduced dramatically, as could be observed by FFT diagrams. 

**Figure 16:**
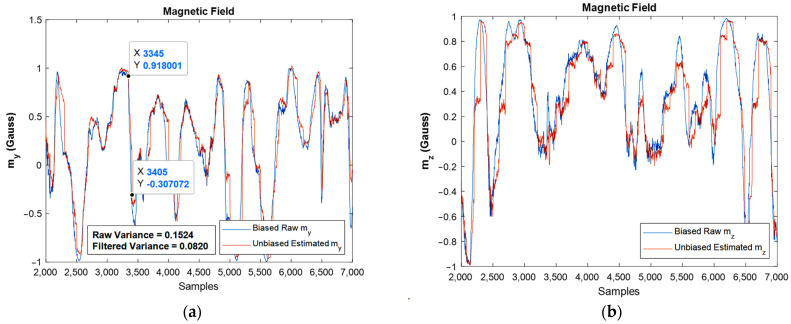
The estimated magnetic field for moving IMU in y and z directions against raw ones: (**a**,**b**), respectively.

The same results were extracted for the magnetometer, and one could observe the estimated magnitudes against the raw one, Figure 16. Again, the fine performance of the developed EKF can be confirmed from the shown plots.

One important point in the Kalman filtering is that in the algorithm, the n’th iteration uses the n-1′th iteration’s result, i.e., the n-1′th iteration contains the effects of the first iteration until n-1′th iterations. As a result, by going forward in the simulation, the results are usually more reliable when compared to the prior ones. However, one of the frequent situations is divergence, which did not occur in the present study. Moreover, variance reduction leads to an increase in the precision of the estimation, and if the noise is Gaussian, the proper Kalman filter minimizes the mean square error of the estimation. The reduction in variance magnitude after filtering is revealed in the moving accelerometer and magnetometer graphs, Figure 15b and Figure 16a, respectively. 

Another contribution of this study is the consideration of gyroscope, accelerometer, and magnetometer biases in the real-time algorithm, in addition to magnetic disturbance effects, simultaneously. Hence, the obtained angular velocities, linear accelerations, and magnetic fields are biasedly compensated for both the stationary and moving IMU. In addition, in all the depicted figures, the scale is considered equal to the better visualization of the extended Kalman filter’s performance.

**Figure 17:**
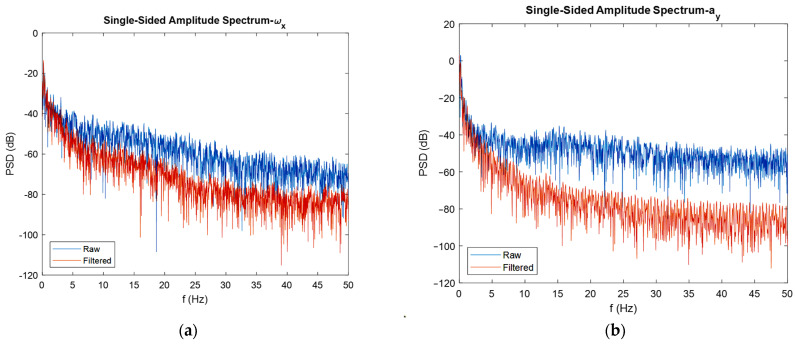

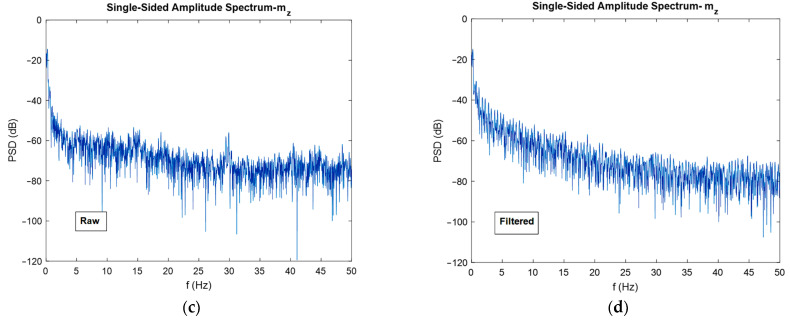
FFT diagrams for moving gyroscope, accelerometer (**a**,**b**), and magnetometer (**c**,**d**).

Now, for discussing the noise cancellation in the moving IMU, the FFT diagrams shown in Figure 17 can be used.

Again, the noise flow was reduced in high frequencies by 10 dB for the gyroscope output. The reduction in noise power for the accelerometer was about 30 dB, and for the magnetometer was almost 5 dB. An interesting observation from the FFT plots built for magnetometers was that the filter eliminated the high-power noises in frequencies that have it. For instance, in about 9, 32, and 42 Hz frequencies, the filter eliminated high power noises very well.

**Figure 18:**
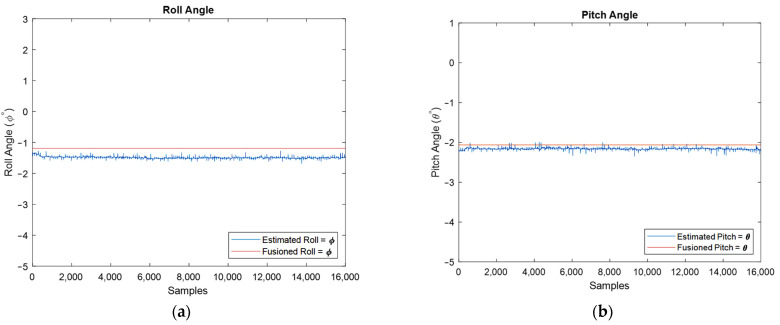

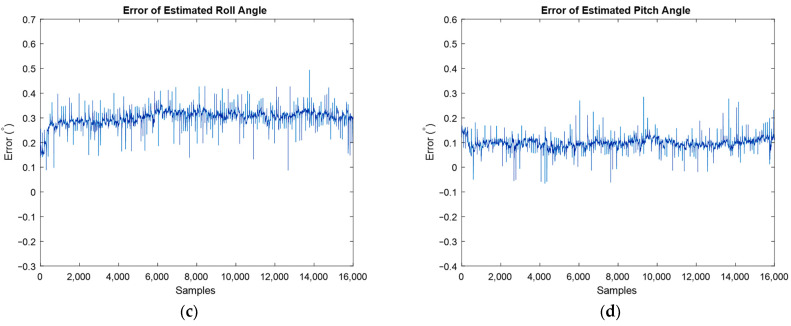
Estimated roll (**a**) and pitch angles (**b**) against filtered IMU output, and estimation error for roll (**c**) and pitch (**d**) angles.

Finally, the used IMU can release two types of data: raw and filtered data. For the discussion of the developed algorithm, the raw data was used to demonstrate the performance of the developed extended Kalman filter. Because the IMU does not provide estimated orientation in its unfiltered mode, i.e., raw measurements, to have a comparison and a benchmark for the developed algorithm, now, the orientation of fusion IMU with the algorithm’s estimated in the stationary mode of IMU is compared for the same conditions, Figure 18. Moreover, the error of estimation angles is depicted using large-scale plots, and, therefore, one can realize that the average error is almost equal to 0.2 degrees. 

**Figure 19:**
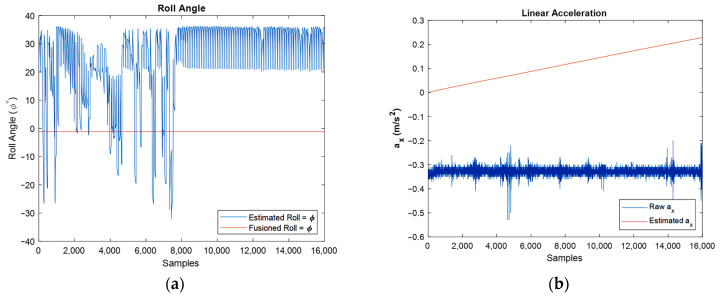

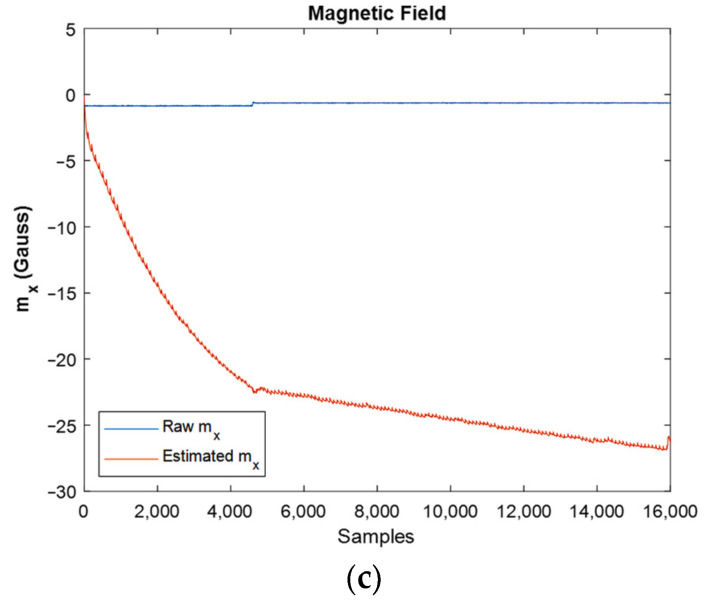
The effect of changing initial conditions in a wrong manner on orientation (**a**), linear acceleration (**b**), and magnetic field (**c**).

An important factor in the systems with nonlinearity is their high sensitivity to initial conditions. In other words, by changing the initial condition even in very small values, the final result will change dramatically. For instance, in Figure 19, one can observe that, although the magnitude of initial orientation has changed a little, the results changed significantly. Particularly, after changing the magnitude of initial quaternions for the stationary IMU case, the results of estimated orientation, linear acceleration, and magnetic field tend to diverge, as it can be found from the shown graphs. In these graphs, the initial quaternion value was changed from proper magnitude values: [0.9998,  0.0108,  0.0184,  0.0000] to the wrong manipulated values: [0.8500,  0.0300,  0.0500,  0.0200].

Here, the orientation estimation, in addition to estimated unbiased angular velocities, linear accelerations, and magnetic fields, was demonstrated for two bunches of IMU data. First, the results were shown for stationary IMU and depicted for a simulation time of 100 s. Then, the results were also presented for moving IMU and a simulation time of 50 s. For evaluation of the developed algorithm, the estimated roll and pitch angles were compared against the filtered ones, and the good performance of the developed EKF’s algorithm can be confirmed. In addition, orientation for the station and moving IMU cases were estimated properly. For instance, in the stationary IMU, the biased angular velocities and linear accelerations were not around the 0 (zero), but this error in the measurements was successfully compensated by the developed algorithm, and the bias was eliminated significantly.

It should be noted that both the integration of a slowly time-varying bias and integration of noise originates from integration drift. Moreover, because the sensor’s bias is different for different axes, this integration drift does not have the same values for all axes. A reduction in the power of noise can be seen in the width of Figure 8 and Figure 9, as well as the FFT diagrams and histograms shown in Figure 11, and Figure 17 shows this reduction clearly. Moreover, the FFT diagrams demonstrated the mean magnitude of the noise power, which means that the assumption about white noise consideration is true. 

For the moving IMU case, the path of angular velocities, linear acceleration, and magnetic fields followed properly after filtering, but the noise and bias were eliminated efficiently. Finally, the effect of changing the initial condition in a wrong manner was investigated and this shows the inherent nonlinearity of the system.

## 6. Conclusions and Future Work

AHRS has a wide application in both academia and industry, and MEMS-based sensors play an important role in this issue. As a result, IMUs are the best selection to reach the accurate orientation of devices in space. Extended Kalman filtering is one of the rigorous probabilistic methods used for orientation estimation when the system is nonlinear.

The focus of this article was on the signal processing aspects of orientation estimation and obtaining unbiased angular velocity, linear acceleration, and magnetic field using IMUs. Thus, the effect of gyroscope and accelerometer biases, as well as the effect of ferromagnetic materials and magnetic disturbances were simultaneously considered. Actually, taking into account all the sensor’s biases as well as the magnetic disturbances is the main contribution of this study. Additionally, the covariance inflation procedure was used to reach smoother results in a two-step EKF algorithm.

It was confirmed that the developed EKF’s algorithm performs properly, and the noise cancellation was tackled as shown in a new manner with the help of useful FFT diagrams. Furthermore, the high sensitivity of the nonlinear system to its initial conditions was confirmed in this study. Filters such as the developed extended Kalman filter use observations up to the time when the state of the dynamic system is to be estimated, but other methods such as smoothing use all measurements to obtain the estimates. Therefore, these methods usually lead to better estimations but are very computationally demanding. However, many practical applications need real-time estimations, and so the developed EKF is very interesting as it can provide accurate orientation estimations in real-time.

In this study, the orientation of IMU was estimated, but estimating the position will be an interesting issue for future work. So, in the next step, it is planned to use vision measurements for position estimation. Moreover, in this study, the experiments were conducted using an IMU and a magnetometer. However, as the next step, the IMU will be combined with a camera to detect its precise orientation and position for image stabilization purpose.

## Data Availability

Not applicable.

## Nomenclature

a	Linear Acceleration
δ	Dip Angle
η	Rotation Vector
ϕ	Roll Angle
g	Earth’s Gravity
Γ	Weakening Effects
K	Kalman Gain
λ	Correlation Time Factor
m	Earth’s Magnetic Field
ω	Angular Velocity
P	State Covariance
Q	Process Noise Covariance
$q_{t}^{nb}$	Unit Quaternion
R	Measurement Noise Covariance
$R_{t}^{nb}$	Rotation Matrix
S	Innovation Matrix
θ	Pitch Angle
v	Measurement White Noise
$w_{t}$	Process Noise
x	Bias
$x_{t}$	System States
$y_{t}$	Measurement
ψ	Yaw Angle

## Abbreviations

The following abbreviations are used in this article:

AHRS	Attitude and Heading Reference System
CI	Covariance Inflation
DOF	Degree of Freedom
EKF	Extended Kalman Filter
FFT	Fast Fourier Transform
IMU	Inertial Measurement Unit
MEMS	Microelectromechanical Systems
SFA	Sensor Fusion Algorithm

## Appendix A. Quaternion Notation and Different Parameterizations

Some of the relations and equations regarding the quaternion issue were mentioned in the article, but written here are other important equations which are also used throughout.

(A1) $$p⊙q=\left(\begin{array}{c} p_{0}\;q_{0}-p_{v}^{T}\cdot q_{v}\\ p_{0}\;q_{v}+q_{0}\;p_{v}+p_{v}\times q_{v} \end{array}\right)=p^{L}q=q^{R}p$$

(A2) $$p^{L}≜\left(\begin{array}{cc} p_{0} & -p_{v}^{T}\\ p_{v} & p_{0}I_{3}+\left[p_{v}\times \right] \end{array}\right),\;q^{R}≜\left(\begin{array}{cc} q_{0} & -q_{v}^{T}\\ q_{v} & q_{0}I_{3}-\left[q_{v}\times \right] \end{array}\right)$$

The notation ⊙ which is used in the article denotes quaternion multiplication.

The matrix vector product form of the cross product can be written as:

(A3) $$u\times v=\left[u\times \right]v=-\left[v\times \right]u\;\;\left[u\times \right]≜\left(\begin{array}{ccc} 0 & -u_{3} & u_{2}\\ u_{3} & 0 & -u_{1}\\ -u_{2} & u_{1} & 0 \end{array}\right)$$

The rotation matrix can be converted or achieved from Euler angles, quaternion notation, and rotation vectors. In this regard, the following relations are important:

(A4) $$\begin{array}{ll} R^{u\;v} & =\;\left(\begin{array}{ccc} \cos\theta \;\cos\psi & \cos\theta \;\sin\psi & -\sin\theta \\ \sin\phi \;\sin\theta \;\cos\psi -\cos\phi \;\sin\psi & \sin\phi \;\sin\theta \;\sin\psi +\cos\phi \;\cos\psi & \sin\phi \;\cos\theta \\ \cos\phi \;\sin\theta \;\cos\psi -\sin\phi \;\sin\psi & \cos\phi \;\sin\theta \;\sin\psi -\sin\phi \;\cos\psi & \cos\phi \;\cos\theta \end{array}\right)\\ & =\left(\begin{array}{ccc} 2q_{0}^{2}+2q_{1}^{2}-1 & 2q_{1}q_{2}-2q_{0}q_{3} & 2q_{1}q_{3}+2q_{0}q_{2}\\ 2q_{1}q_{2}+2q_{0}q_{3} & 2q_{0}^{2}+2q_{2}^{2}-1 & 2q_{2}q_{3}-2q_{0}q_{1}\\ 2q_{1}q_{3}-2q_{0}q_{2} & 2q_{2}q_{3}+2q_{0}q_{1} & 2q_{0}^{2}+2q_{3}^{2}-1 \end{array}\right) \end{array}$$

Moreover, one can write:

(A5) $$if\;R=\left(\begin{array}{ccc} R_{11} & R_{12} & R_{13}\\ R_{21} & R_{22} & R_{23}\\ R_{31} & R_{32} & R_{33} \end{array}\right)\;\Rightarrow \;q_{0}=\frac{\sqrt{1+Tr\;R}}{2},\;q_{v}=\frac{1}{4q_{0}}\left(\begin{array}{c} R_{32}-R_{23}\\ R_{13}-R_{31}\\ R_{21}-R_{12} \end{array}\right)$$

and for rotation vector format, one has:

(A6) $$\begin{array}{l} \exp\left(\bar{\eta }\right)=\left(\begin{array}{c} \cos{\left\|\eta \right\|}_{2}\\ \frac{\eta }{{\left\|\eta \right\|}_{2}}\sin{\left\|\eta \right\|}_{2} \end{array}\right)\\ \exp\left(\left[\eta \times \right]\right)=I_{3}+\sin\left({\left\|\eta \right\|}_{2}\right)\left[\frac{\eta }{{\left\|\eta \right\|}_{2}}\times \right]+\left(1-\cos\left({\left\|\eta \right\|}_{2}\right)\right){\left[\frac{\eta }{{\left\|\eta \right\|}_{2}}\times \right]}^{2} \end{array}$$

Then, the Euler angles can be written as:

(A7) $$\begin{array}{l} \psi =\tan^{-1}\left(\frac{R_{12}}{R_{11}}\right)=\tan^{-1}\left(\frac{2q_{1}q_{2}-2q_{0}q_{3}}{2q_{0}^{2}+2q_{1}^{2}-1}\right)\\ \theta =-\sin^{-1}\left(R_{13}\right)=-\sin^{-1}\left(2q_{1}q_{3}+2q_{0}q_{2}\right)\\ \phi =\tan^{-1}\left(\frac{R_{23}}{R_{33}}\right)=\tan^{-1}\left(\frac{2q_{2}q_{3}-2q_{0}q_{1}}{2q_{0}^{2}+2q_{3}^{2}-1}\right) \end{array}$$
